# Learning from maternal deaths due to uterine rupture: review of cases from peri-urban Uganda

**DOI:** 10.1016/j.xagr.2022.100063

**Published:** 2022-06-11

**Authors:** Imelda Namagembe, Sarah M. Chukwuma, Annettee Nakimuli, Noah Kiwanuka, Josaphat Byamugisha, Ashley Moffett, Catherine E. Aiken

**Affiliations:** a Department of Obstetrics and Gynaecology, Makerere University and Mulago National Referral Hospital, Kampala, Uganda (Drs Namagembe, Nakimuli, and Byamugisha); b Department of Obstetrics and Gynaecology, James Paget University Hospitals NHS Foundation Trust, Great Yarmouth, United Kingdom (Ms Chukwuma); c Department of Epidemiology and Biostatistics, School of Public Health, Makerere University, Kampala, Uganda (Dr Kiwanuka); d Department of Pathology and Centre for Trophoblast Research, University of Cambridge, Cambridge, United Kingdom (Dr Moffett); e Department of Obstetrics and Gynaecology, University of Cambridge, Rosie Hospital and National Institute for Health and Care Research Cambridge Biomedical Research Center, Cambridge, United Kingdom (Dr Aiken).

**Keywords:** birth preparation, cesarean delivery, maternal death, obstructed labor, partograph, sub-Saharan Africa, Uganda, uterine rupture

## Abstract

**BACKGROUND:**

Maternal deaths from uterine rupture continue to occur globally, with particularly high rates in sub-Saharan Africa. Maternal death reviews have been shown to be an effective part of cohesive strategies to prevent future deaths.

**OBJECTIVE:**

This study aimed to conduct maternal death reviews for all deaths following uterine rupture in the study center, to assess preventability, and to synthesize key learning points that may help to prevent future maternal deaths following uterine rupture.

**STUDY DESIGN:**

Thorough case reviews of all maternal deaths from 2016 to 2018 at the study center (a national referral hospital in urban Uganda) were conducted by trained multidisciplinary panels of obstetricians and midwives. Medical records of women who died following uterine rupture (n=37, 10.6% of all maternal deaths) were extracted for further analysis.

**RESULTS:**

Most maternal deaths due to uterine rupture (36/37, 97%) were preventable, with most having been still potentially preventable after the women reached the study center (24/36, 67%). Obstructed labor was the leading cause of uterine rupture, accounting for 73% (27/37) of cases. Previous cesarean delivery was confirmed in 38% (14/37) of cases. The incidence of grand multiparity was 11% (4/37), and 11% (4/37) were primiparous. Most women (28/37, 76%) died within 24 hours of admission. On arrival at the study center, 19 (51%) were critically ill. Exploratory laparotomy was performed in 54% (20/37) of cases, and a further 35% (13/37) died while awaiting laparotomy. Four women died shortly after arrival at the study center (within 1 hour) and received basic resuscitative treatment; 27% (10/37) of women who died had received antenatal planning or preparation.

**CONCLUSION:**

Most deaths due to uterine rupture were preventable. The key lessons that emerged from the reviews were: (1) careful birth preparation and complication awareness for women with known risk factors, (2) early recognition of obstructed labor, (3) close monitoring of obstetrical interventions known to be associated with uterine rupture, and (4) treating incipient or suspected uterine rupture as a time-critical obstetrical emergency. The recommendations emerging from our narrative reviews are suitable for implementation in low-resource obstetrical settings, where high numbers of deaths involving uterine rupture occur.

**AJOG Global Reports at a Glance**

**Why was this study conducted?**

This study aimed to conduct detailed maternal death reviews for cases of uterine rupture in a low-resource obstetrical setting and generate learning points.

**Key findings**

Obstructed labor was the leading cause of uterine rupture in this setting. Careful birth preparation, early recognition of obstructed labor, close monitoring, and time-critical treatment is recommended to prevent death due to uterine rupture.

**What does this add to what is known?**

What does this add to what is known?

Most deaths from uterine rupture are potentially preventable with prompt and adequate management. The outlined learning points are not resource-intensive and are thus suitable for application in a low-resource obstetrical setting.

## Introduction

Uterine rupture carries a high burden of maternal mortality, primarily owing to catastrophic bleeding into the abdomen. As rates of cesarean delivery and use of oxytocin for augmentation or induction of labor increase worldwide, rates of uterine rupture have also been increasing.^1^ It is thus of key importance to identify strategies to prevent death in cases of uterine rupture. We present a uniquely large case series of maternal deaths from uterine rupture in a low-resource obstetrical setting, from which we draw learning points.

Estimates of maternal case fatality from uterine rupture in sub-Saharan African settings range from 2.2% to 12%,^2^, ^3^, ^4^ compared with approximately 1% in the United Kingdom.^5^ Estimates of neonatal mortality following complete uterine rupture vary from 69% to 75% in low-resource obstetrical settings,^2^^,^^4^ compared with 12% to 25% in better-resourced settings.^6^

Conducting detailed case reviews of maternal deaths is an important intervention recommended by the World Health Organization^7^ and has been demonstrated to reduce mortality across different global settings. However, there are few obstetrical settings in which robust maternal death review is routinely performed, and sufficient cases are available to study rarer causes of maternal death, such as uterine rupture. For example, a year-long study conducted in the United Kingdom identified only 2 deaths from uterine rupture,^5^ and a 2-year study in Belgium identified no maternal deaths from 90 cases of uterine rupture.^8^ There is a similar scarcity of robust data from resource-limited obstetrical settings.^3^^,^^4^

Our study was conducted at a national obstetrics referral center in Kampala, Uganda, which is the largest maternity unit in sub-Saharan Africa.^9^^,^^10^ Our in-depth and comprehensive data collection allowed us to present a uniquely large case series of maternal deaths from confirmed or clinically suspected uterine rupture. The rate of maternal death from uterine rupture (>1 per month throughout the study period) allowed us to identify themes and key messages from the narrative summaries of maternal deaths. We aim to highlight learning points and recommendations that may help reduce maternal death rates from uterine rupture in similar settings.

## Materials and Methods

A comprehensive review of all maternal deaths occurring in a single tertiary obstetrics center in Uganda was performed (January 2016–December 2018). The study center accepts referrals from all areas of Uganda, in addition to providing publically funded care for local women, and accommodates approximately 25,000 to 30,000 deliveries annually. On average, 2 to 3 maternal deaths per week occur at the study center. We identified all maternal deaths in the relevant time frame by cross-checking maternity admissions with mortuary records for women of reproductive age (n=401), excluding any who did not meet the study definition of maternal death occurring within 42 days after end of pregnancy (n=350 maternal deaths).^11^

The analytical sample includes all women who died following a diagnosis of uterine rupture (n=37/350, 10.6%). Postmortem examination is rarely performed in Uganda because it is resource-intensive, expensive, and must be approved by next-of-kin. It was therefore not possible to confirm all cases of uterine rupture after death. We thus accepted a diagnosis of uterine rupture on any of the following grounds: (1) confirmed at postmortem, (2) confirmed at laparotomy, and (3) strongly suspected by clinicians, with evidence recorded in the medical notes (eg, fetal parts felt abdominally), but death occurred before operative intervention.

All case notes were identified from the institutional records and retrieved by the study team. The full anonymized medical records were reviewed by local multidisciplinary review panels convened for this specific purpose. All clinicians received study-specific training in maternal death review and signed confidentiality agreements before reviewing any cases. The specific composition and training of these panels is detailed elsewhere.^11^

The review panels produced detailed narrative outcomes for each case and categorized each case according to key medical and demographic characteristics. All panels were asked to reach a consensus opinion on the preventability of each death and to record what specific measures might have prevented death. Additional opinions on these aspects were sought from other expert local clinicians where appropriate. Each case summary was further reviewed independently by an international obstetrician who was familiar with local contextual factors, for quality and completeness.

We present summary statistics for all cases where uterine rupture occurred before maternal death. Data are presented as number (percentage) or median where appropriate. Small-number suppression was applied to demographic data where relevant. We also present anonymized case narratives to illustrate important messages. The key elements of each narrative were preserved; however, some details have been changed to preserve anonymity.

The study was approved by the Makerere University School of Medicine Research and Ethics Committee (SOMREC; #REC Ref 2018-001) and by the Uganda National Council for Science and Technology (UNCST; Ref SS4797).

## Results

### Demographic details

A total of 37 women died following uterine rupture (median age, 28 years; range, 20–45 years); 19 (51%) women were full-term (≥37 weeks of gestation) at the time of death. Twenty-nine (78%) women were married (Table 1).

**Table 1:** Demographic characteristics of mothers who died of complications of uterine rupture (2016–2018)

Maternal characteristic		Frequency (n=37)	Proportion (%)
Age (y)	20–34	28	75.7
	≥35	9	24.3
Marital status	Married	29	78.4
	Unmarried/unknown	8	21.6
Distance traveled	<10 km	12	32.4
	≥10 km	25	67.6
Referral status	Referred	29	78.4
	Not referred	8	21.6
Parity	0	4	10.8
	1–4	29	78.4
	≥5	4	10.8
Gestation	28–36 wk	4	10.8
	≥37 wk	19	51.4
	Unknown	14	37.8
Previous cesarean delivery	Yes	14	37.8
	No	8	21.6
	Unknown	15	40.5
Antenatal care	Yes	10	27.0
	No or unknown	27	73.0

The median number of previous deliveries was 3, with a range of 0 to 12. Four (11%) women were grand multiparous (≥5 previous deliveries) and another 4 (11%) were primiparous. Fourteen (38%) women were recorded as having had a previous cesarean delivery. In 8 (22%) cases, no previous uterine surgery had occurred, but this information was incomplete for 15 (41%) others. In total, 111 children were left motherless by the deaths of these 37 women.

In 27 (73%) cases, a diagnosis of obstructed labor was made. In 18 (49%) cases, the diagnosis of uterine rupture was made at laparotomy, another 17 (46%) cases were diagnosed contemporaneously on clinical grounds, and 2 (5%) cases were confirmed at postmortem examination (Table 1).

### Narrative 1: Obstructed labor in primiparous women

Patricia was a 40-year-old married woman who lived in the suburbs of Kampala. She was pregnant with her first child at full-term. She had not sought any antenatal care. Patricia began labor unattended at home, but after several days attended her local healthcare facility. At the clinic, she was found to be anemic, dehydrated, and exhausted. Shortly after attending, she collapsed and was transferred to the study center. On arrival, she was unconscious and in critical condition. A peritonitic abdomen and hematuria were evident on examination. Patricia underwent a perimortem cesarean delivery within minutes of arrival at the study center, revealing a ruptured uterus with a large hemoperitoneum and a recently stillborn infant. Patricia was transfused 1 unit of blood and a hysterectomy was attempted, but she could not be adequately resuscitated and sadly died intraoperatively.

### Accessing medical care

Ten (27%) women were recorded as having received antenatal care (median 3 visits; range, 1–5). The remaining 27 (73%) either did not receive any antenatal care or records were not available.

The maximum distance traveled by women to the study center was approximately 300 km, and the median distance traveled was 30 km. Twenty-nine (78%) women initially presented to another healthcare facility and were subsequently transferred to the study center for escalated care (Table 1).

### Narrative 2: Adequate antenatal planning

Judith was a 40-year-old married woman who lived in the suburbs surrounding Kampala. She had 11 children, all born by normal vaginal delivery. For her twelfth pregnancy, Judith attended 3 antenatal care appointments at the study center. At her last appointment, she was advised to present for review in absence of delivery by 4 weeks after her due date. However, at 40 weeks of gestation, after a short period of mild uterine tightening, Judith experienced sudden profuse vaginal bleeding and abdominal pain. She attended her local clinic and was swiftly referred to the study center, where she arrived in critical condition with an unrecordable blood pressure. On examination, fetal parts were easily palpable abdominally. Uterine rupture was suspected, and a plan was made for an emergency laparotomy. She had ongoing heavy vaginal bleeding and was transfused 1 unit of blood. Sadly, Judith died undelivered within 40 minutes of arrival at the study center.

### Condition on admission

Survival time from admission ranged between 2 minutes and 23 days (Table 2). The median admission duration at the study center before death was 3 hours and 17 minutes. Most women (n=28; 76%) died within 24 hours of admission. On arrival at the study center, 19 (51%) of the women were judged by the admitting clinical team to be already critically ill. Among these women, 9 (47%) died within 60 minutes, and 6 (32%) within 60 to 120 minutes.

**Table 2:** Clinical characteristics of the mothers who died of complications of uterine rupture (2016–2018)

Maternal characteristic			Frequency (n=37)	Proportion (%)
Clinical condition on arrival	Not critically ill		18	48.6
	Critically ill		19	51.4
Basis of diagnosis	Laparotomy		18	48.6
	Clinical grounds		17	45.9
	Postmortem		2	5.4
Time from admission to death	Not critically ill on arrival	<2 h	3	16.7
		2–24 h	8	44.4
		>24 h	7	38.9
	Critically ill on arrival	<2 h	15	79.0
		2–24 h	2	10.5
		>24 h	2	10.5
Delay in care	Yes		27	73.0
	No		10	27.0
Blood pressure recorded	Yes		26	70.3
	No/unknown		11	29.7
Respiratory rate recorded	Yes		8	21.6
	No/unknown		29	78.4
Neonatal status	Survived		2	5.4
	Died		18	48.6
	Not delivered		17	45.9

Twenty-six (70%) women had their blood pressures taken on arrival at the hospital, of whom 10 (27%) had critically low (≤90/60) or unrecordable blood pressure.

Seventeen (46%) women died before delivery. From the entire cohort of 37 women, only 2 (5%) infants were born alive. Most delivered infants were classified as intrapartum stillbirths (14/20; 70%).

### Treatments

Exploratory laparotomy was performed in 20 (54%) cases. Another 13 (35%) women died awaiting laparotomy. The remaining 4 (11%) women died within 60 minutes of arrival and received only basic resuscitative treatment (Table 2). Of those who were operated on, 13 (65%) had a hysterectomy. Six (30%) of the women who were operated on died intraoperatively or shortly after transfer to the intensive care unit.

Fourteen (38%) women received a blood transfusion. Four (11%) women received antibiotics during their admission, and another 2 cases mention sepsis; however, no antibiotic use was documented.

Three (8%) women had their labors augmented with oxytocin. Of the 31 (84%) women who labored, none had their labors monitored with a partograph.

### Preventability and delays in care

The review panels judged that all but 1 of the maternal deaths due to uterine rupture were preventable (36/37; 97%). Delay in seeking medical care at the individual or family level accounted for 14 (39%) of these cases. Eight (22%) women were delayed at another health facility because of reasons such as lack of facilities or staff.

Of the women whose deaths were deemed preventable, 67% (24/36) experienced delay after arrival at the study center. The review panel judged that there was an avoidable delay in 13 (35%) cases due to lack of blood, supplies, or consumables (Table 3). This included operating space, blood for transfusion, surgical instruments, and medicines.

**Table 3:** Treatments received by mothers who died of complications of uterine rupture (2016–2018)

Maternal characteristic		Frequency (n=37)	Proportion (%)
Laparotomy	Yes	20	54.1
	No – died awaiting	13	35.1
	No – basic resuscitation	4	10.8
Blood transfusion	Yes	14	37.8
	No	23	62.2
ICU care	Yes	2	5.4
	No/unknown	35	94.6
Antibiotics	Yes	4	10.8
	No/unknown	33	89.2
Augmentation of labor	Yes	3	8.1
	No/unknown	34	91.9

ICU, intensive care unit.

### Narrative 3: Delay in accessing laparotomy and clinical prioritization

Leila was a 28-year-old quadripara, having had 4 previous normal deliveries. Leila attended a local clinic (>50 km from the study center) in active labor, where she progressed well to full dilatation. Unfortunately, a diagnosis of face presentation was made and she was transferred to the study center for cesarean delivery. Leila arrived at the study center in the early hours of the morning, where the diagnosis of obstructed labor and plan to operate were confirmed. However, because of lack of supplies and staff there was a delay in accessing operating facilities. Sadly, Leila collapsed while waiting and died, just over 7 hours from admission.

Women were equally likely to die on weekdays compared with weekends. More women died outside of regular working hours: 8 women died during regular working hours (average, 0.9 deaths/hour), compared with 29 women who died outside of working hours (average, 1.9 deaths/hour; *P*<.05), when specialist obstetricians are not routinely available at the study center.

### Narrative 4: Early involvement of specialist doctors

Betty was a 35-year-old single woman who lived in urban Kampala. She had 4 children, 3 of whom were delivered via cesarean delivery. Betty was diagnosed antenatally with placenta previa and was advised to attend for review if she experienced vaginal bleeding. She presented at 35 weeks of gestation in preterm labor with extensive vaginal bleeding. The admitting doctor made a plan for an emergency cesarean delivery. On opening the abdomen, a recently stillborn 3-kg infant was delivered, and a ruptured uterus was diagnosed. The admitting doctor then attempted a subtotal abdominal hysterectomy; however, the bleeding was difficult to control, and the operation was technically challenging. Suitable blood products were not available. It was by now late in the evening, and no specialist obstetrician was immediately available or called into the hospital. Betty sadly died 4 hours after being admitted to the study center of intraoperative cardiac arrest due to blood loss.

## Comment

We present the findings of a large case series of women who died from uterine rupture with the key aim of deriving implementable recommendations from these tragic outcomes. It is particularly important to highlight lessons from these maternal deaths given the increasing prevalence of risk factors for uterine rupture in maternity populations worldwide.^12^

### Principal findings and results

Most deaths from uterine rupture occurred within a short time frame after reaching the study center (median, 3.3 hours). When uterine rupture is suspected or diagnosed, it is a true obstetrical emergency. Emergency laparotomy must be prioritized to achieve a realistic chance of saving the mother's life. Despite prompt surgical intervention in many of our reported cases, many mothers were moribund on arrival and could not be resuscitated. To facilitate prevention, there must be a focus on: (1) suitable delivery planning and (2) early recognition of obstructed labor. The high proportion of women in our study who were transferred from other healthcare facilities (78%) highlights the importance of avoiding delay in definitive care for preventing death due to uterine rupture.

### Clinical implications

#### Adequate antenatal planning for high-risk women

Many of the causes of uterine rupture are well-described.^1^^,^^3^^,^^13^^,^^14^ In particular, the presence of a previous uterine scar is a basic and crucial piece of information for any obstetrical caregiver. The significant percentage of mothers who died (41%) where this information was not known highlights that this is a key point of medical history-taking that was underrecorded.

Women of higher parity are thought to be more vulnerable to uterine rupture than primiparous women because repeated term pregnancy may result in fibrosis and thinning of the myometrium.^15^ This may be especially problematic if the interpregnancy interval is short (<16 months), as is common in obstetrical settings with limited access to contraception. Although grand multiparity (11% of our cohort) is not common and is decreasing worldwide,^16^ it remains an important risk factor for adverse outcomes. Therefore, it is essential to provide these women with adequate counseling antenatally about the management of prolonged pregnancy.

#### Early recognition of obstructed labor

Unassisted or unmonitored labors are common in women for whom there are significant barriers to accessing medical care. Lack of monitoring during labor increases the risk that obstructed labor will go unrecognized^17^ and increases the likelihood of delay in transfer to medical settings.^18^ Our study highlights that alertness for clinical signs of obstructed labor is imperative.

Previous evidence from other contexts suggests that use of a partogram or Labor Care Guide is a helpful adjunct to improve the recognition of obstructed labor.^17^ This is a low-cost intervention suitable for implementation in almost all settings that is recommended by the World Health Organization.^19^, ^20^, ^21^

#### Close monitoring of interventions known to be associated with uterine rupture

Women who receive excessive oxytocin or prostaglandins during labor or attempts to induce labor may be vulnerable to uterine rupture,^22^ highlighting the importance of caution and monitoring when these interventions are used.

Globally, the rate of cesarean delivery is escalating.^23^^,^^24^ Although cesarean delivery is a life-saving intervention, it is also associated with long-term maternal risks for future pregnancies including uterine rupture.^25^ Sub-Saharan Africa has the lowest cesarean delivery rate worldwide; however, this is slowly rising.^26^ The rise is attributed to the increased number of maternal requests for cesarean delivery,^27^ and an evolving pattern of increased maternal and fetal risk.^28^

#### Treat suspected or incipient rupture as an obstetrical emergency

We show that the average arrival to death interval in cases of uterine rupture was a few hours. Saving maternal lives in the context of suspected uterine rupture is therefore time-critical. Prioritization of women who have clear risk factors for rupture as emergencies for operative intervention is a clear message that emerges from our data. The average time to cesarean delivery in this setting is strikingly long when assessed against international standards^29^^,^^30^; however, this is not unusual compared with similar low-resource settings.^31^^,^^32^ Our finding of an increased rate of death from uterine rupture outside of normal working hours underscores the importance of obtaining support from the most experienced obstetrician available at an early stage.

### Research implications

An important future research goal is to collate similar case series of maternal deaths in other obstetrical settings. This will help to determine which causes and learning points are context-specific and which can be more widely generalized to other settings. Our findings have implications for both expansion of antenatal planning and modification of intrapartum care to emphasize early recognition of obstructed labor.

### Strengths and limitations

The key strength of our analysis is that we have assembled a large case series of deaths from uterine rupture,^33^^,^^34^ using a systematic and comprehensive approach to identifying maternal deaths over a 3-year period.^11^ In the context of rising case numbers of uterine rupture worldwide,^35^ review of the learning points from these deaths is both timely and important.

An important feature of our study is that it was performed in a single obstetrics center in Kampala, and hence the results may not be readily generalizable to other settings. However, this is somewhat mitigated by the fact that the study center is one of the busiest obstetrical units in Africa (25,000–30,000 deliveries annually), and the complete dataset of maternal deaths from all causes (n=350) includes women who lived in 27 of 135 districts of Uganda, with residences ranging from 0.6 to 433 km from the study center.

### Conclusions

Evidence from maternal death reviews specific to uterine rupture is scarce, making learning points and recommendations difficult to derive. We highlighted the importance of prompt-care and identified that delays at home, during transfer, and in health facilities^36^ all contribute to deaths from uterine rupture. Our key recommendations include: (1) careful birth preparation and complication awareness for women with known risk factors, (2) early recognition of obstructed labor followed by timely intervention likely to reduce uterine rupture, (3) close monitoring of interventions known to be associated with uterine rupture, and (4) treating suspected uterine rupture as a time-critical obstetrical emergency.
